# Supplementary material for: Plasma proteome profiling discovers novel proteins associated with non‐alcoholic fatty liver disease
Source: Mol Syst Biol. 2019 Mar 1;15(3):e8793. doi: 10.15252/msb.20188793 (PMC6396370; doi:10.15252/msb.20188793)
Supplement: Supplementary file 1 — Expanded View Figures PDF [file MSB-15-e8793-s001.pdf]

Expanded View Figures

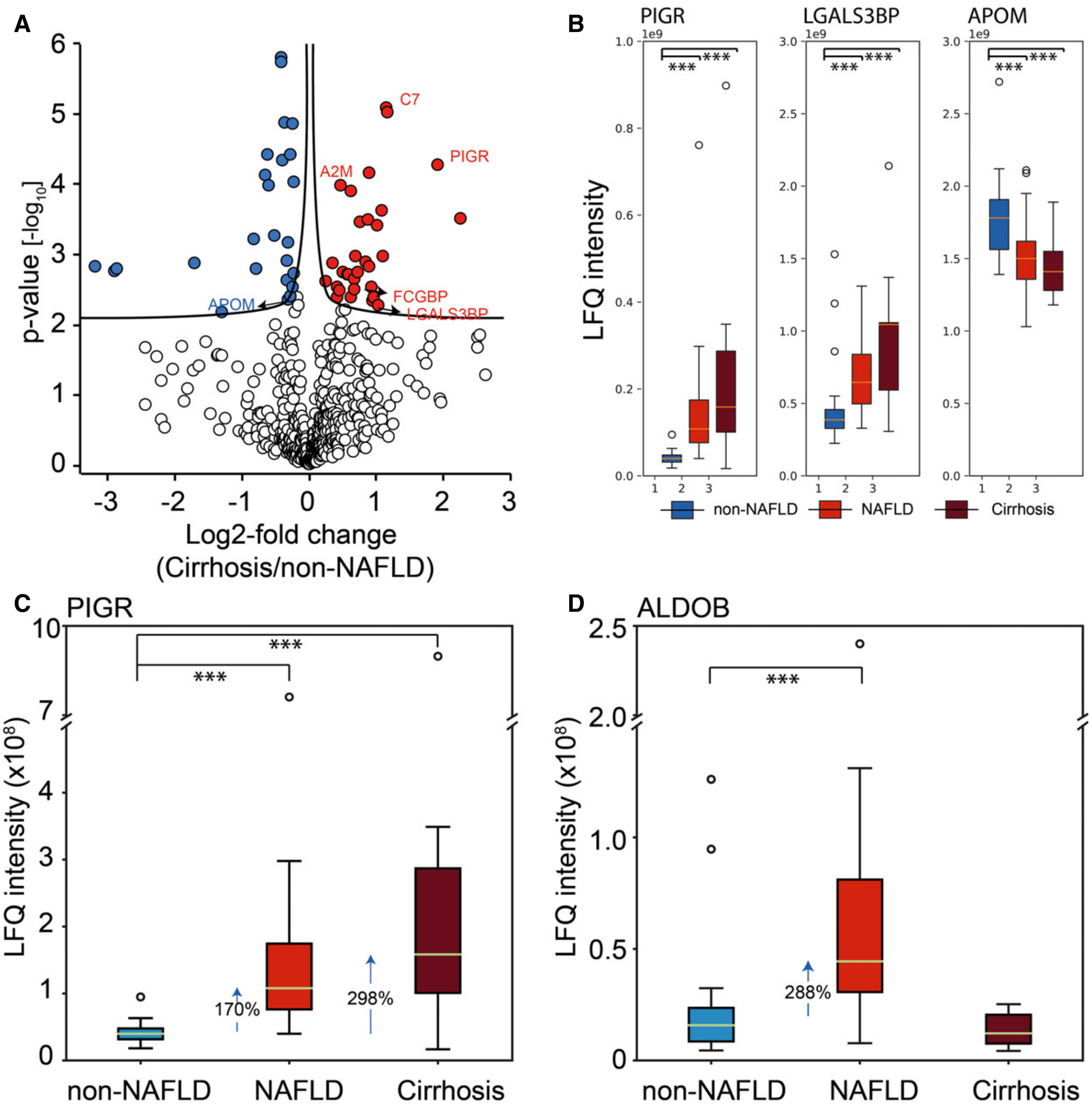

Figure EV1. Overlapping significantly regulated proteins in NAFLD and cirrhosis.

A Volcano plot of statistical significance against log2-fold change between the non-NAFLD group and the cirrhosis group. Significance is controlled by *P*-value and minimum fold change (s0 parameter in the Perseus software) indicated by the cutoff curve, highlighting proteins overlapped with two marker panels for NAFLD subtypes.

B Box-and-whisker plot showing the distribution of LFQ intensity values of PIGR, LGALS3BP, and APOM in plasma. Number of replicates are 18, 20, and 10 in non-NAFLD, NAFLD, and Cirrhosis, respectively. The yellow line is the median, the top and the bottom of the box represent the upper and lower quartile values of the data and the whiskers represent the upper and lower limits for considering outliers (Q3+1.5*IQR, Q1-1.5*IQR) where IQR is the interquartile range (Q3–Q1). ***, $P < 0.001$. (independent two-sample *t*-test, two-sided).

C Box-and-whisker plot showing the distribution of LFQ intensity values of PIGR in plasma with median fold change indicated. Number of replicates in each group and representation of boxes and whiskers are defined as in panel (B).

D Box-and-whisker plot showing the distribution of LFQ intensity values of ALDOB in with median fold change indicated. Number of replicates in each group and representation of boxes and whiskers are defined as in panel (B).

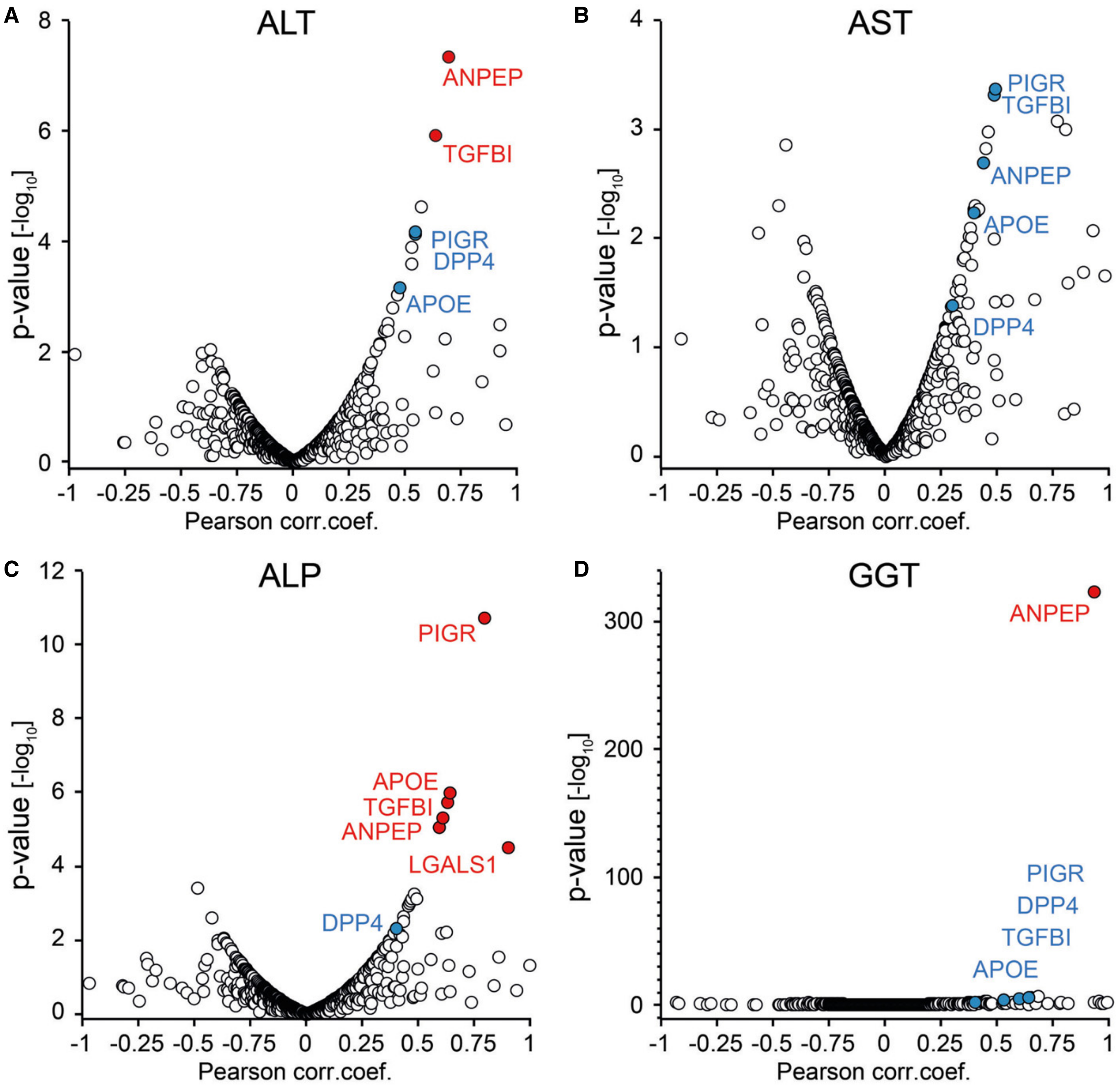

Figure EV2. Pearson correlation of all proteins to individual liver enzymes.

A–D Four liver enzymes are ALT, AST, ALP, and GGT, and red color-coded proteins are significant.

 © 2019 The Authors

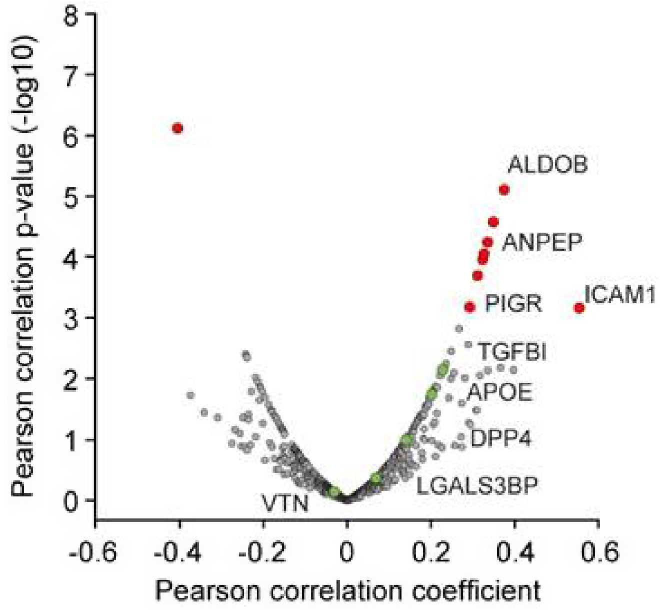

Figure EV3. Pearson correlation of plasma proteins to ALT in a separate dataset.

This dataset has been published in Wewer Albrechtsen *et al* (2018) as described in the main text. Red color-coded proteins are significant.

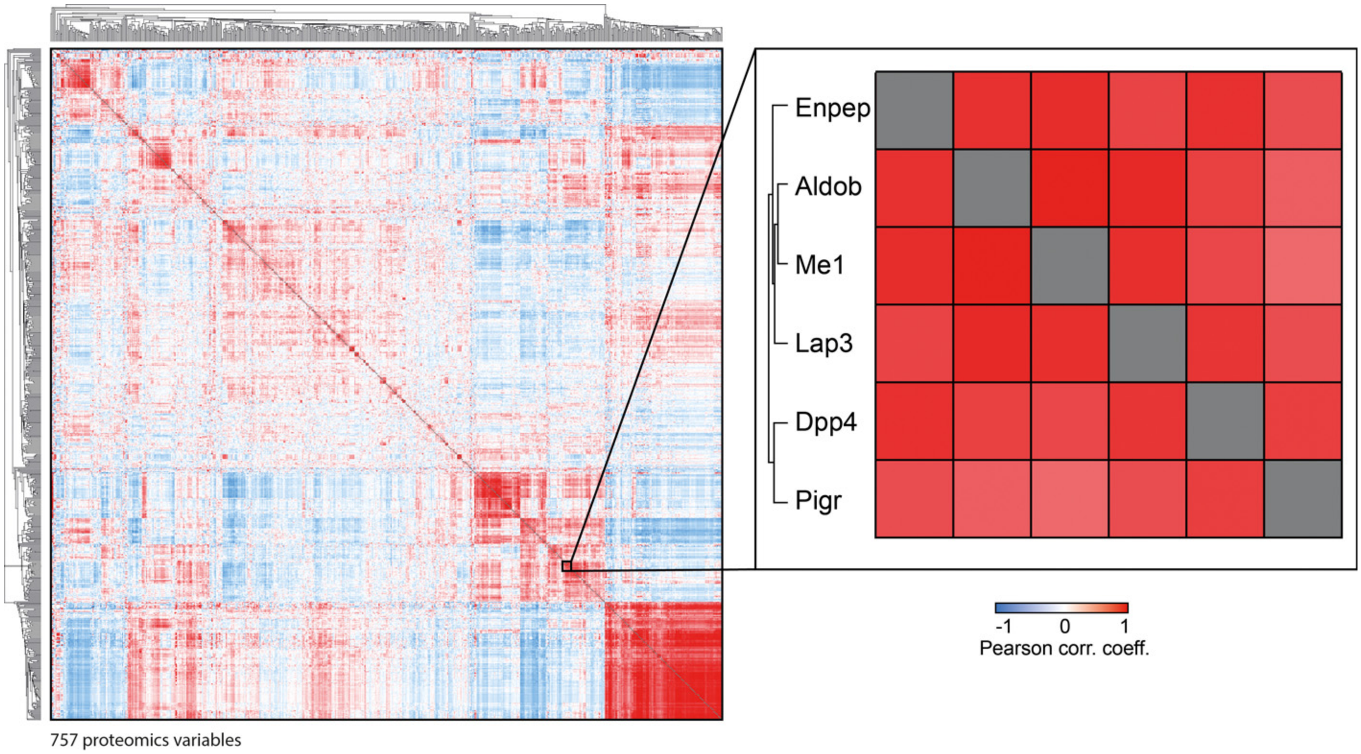

Figure EV4. Protein–protein correlation map of mouse plasma proteome.

Pairwise correlation of plasma proteins over 82 mice, resulting in a matrix of correlation coefficients where each variable is compared to all others. Variables with a high positive correlation with each other will cluster together in groups of red rectangles. Negative correlation is indicated in blue patches. The magnified area highlights a cluster of six proteins of special interest that co-vary under pathophysiological conditions.
