# Supplementary material for: Plasma proteome profiling discovers novel proteins associated with non‐alcoholic fatty liver disease
Source: Mol Syst Biol. 2019 Mar 1;15(3):e8793. doi: 10.15252/msb.20188793 (PMC6396370; doi:10.15252/msb.20188793)

Table EV1: One-way ANOVA with multiple hypothesis testing corrected by Benjamini-Hochberg at a level below 0.05. Six of eight statistically significantly different proteins in the two NAFLD cohorts tested by two-sided independent t-test have statistically significantly different means across all five groups in the human study.


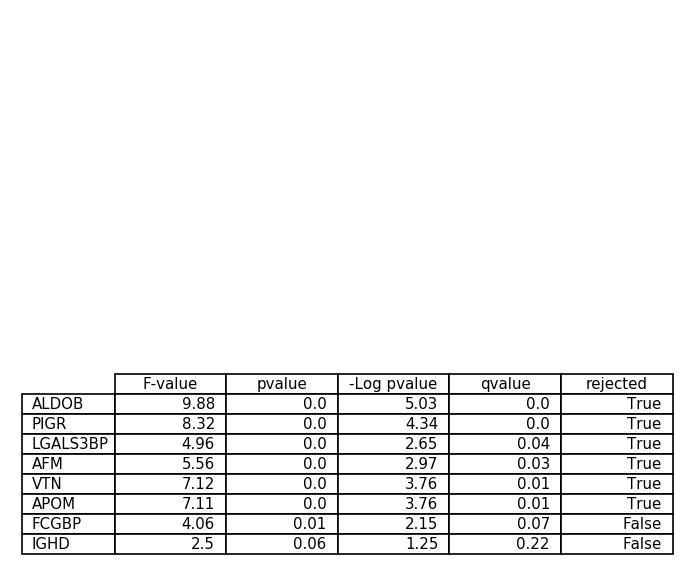

Supplement: Supplementary file 2 — Table EV1 [file MSB-15-e8793-s002.docx]
